# Supplementary material for: Active site specificity profiling datasets of matrix metalloproteinases (MMPs) 1, 2, 3, 7, 8, 9, 12, 13 and 14
Source: Data Brief. 2016 Feb 22;7:299–310. doi: 10.1016/j.dib.2016.02.036 (PMC4777984; doi:10.1016/j.dib.2016.02.036)
Supplement: Supplementary file 10 — Supplementary material [file mmc10.zip › WebPICS_hMMP3_T_1%/P3.html]

 

PICS results


|  |  |
| --- | --- |
| **P3\_A**  24 in 199 sites   12.1 %    effects > 10 perc. pnts.  (vice-versa in brackets)  P1\_N: 12.9 (12.9)   P1prime\_I: 18.2 (14.6)   P3prime\_Q: 10.7 (21.2) |  |
| **P3\_C**  3 in 199 sites   1.5 %    effects > 10 perc. pnts.  (vice-versa in brackets)  P2prime\_I: 88.9 (12.1) |  |
| **P3\_H**  6 in 199 sites   3.0 %    effects > 10 perc. pnts.  (vice-versa in brackets)  P1\_Q: 27.8 (15.2)   P2\_L: 39.4 (11.3)   P2\_W: 14.7 (22.0)   P2prime\_Q: 27.8 (15.2) |  |
| **P3\_I**  17 in 199 sites   8.5 %    effects > 10 perc. pnts.  (vice-versa in brackets)  P1\_D: 18.8 (15.3)   P1prime\_C: 12.6 (21.5)   P3prime\_A: 22.7 (15.5)   P3prime\_E: 10.6 (12.9) |  |
| **P3\_L**  23 in 199 sites   11.6 %    effects > 10 perc. pnts.  (vice-versa in brackets)  P1\_E: 32.1 (18.4)   P2prime\_T: 11.6 (13.4)   P3prime\_Q: 11.4 (21.7) |  |
| **P3\_V**  22 in 199 sites   11.1 %    effects > 10 perc. pnts.  (vice-versa in brackets)  P1\_G: 22.8 (27.8)   P1\_S: 10.2 (13.9)   P2\_G: 14.7 (12.9) |  |
